# Recumbence Behavior in Zoo Elephants: Determination of Patterns and Frequency of Recumbent Rest and Associated Environmental and Social Factors

**DOI:** 10.1371/journal.pone.0153301

**Published:** 2016-07-14

**Authors:** Matthew R. Holdgate, Cheryl L. Meehan, Jennifer N. Hogan, Lance J. Miller, Jeff Rushen, Anne Marie de Passillé, Joseph Soltis, Jeff Andrews, David J. Shepherdson

**Affiliations:** 1 Department of Biology, Portland State University, Portland, Oregon, United States of America; 2 Conservation Research Division, Oregon Zoo, Portland, Oregon, United States of America; 3 AWARE Institute, Portland, Oregon, United States of America; 4 Chicago Zoological Society—Brookfield Zoo, Brookfield, Illinois, United States of America; 5 Dairy Education and Research Centre, University of British Columbia, Agassiz, Canada; 6 Department of Education & Science, Disney’s Animal Kingdom, Lake Buena Vista, Florida, United States of America; 7 Zoological Operations, Busch Gardens, Tampa, Florida, United States of America; University of Tasmania, AUSTRALIA

## Abstract

Resting behaviors are an essential component of animal welfare but have received little attention in zoological research. African savanna elephant (*Loxodonta africana*) and Asian elephant (*Elephas maximus*) rest includes recumbent postures, but no large-scale investigation of African and Asian zoo elephant recumbence has been previously conducted. We used anklets equipped with accelerometers to measure recumbence in 72 adult female African (n = 44) and Asian (n = 28) elephants housed in 40 North American zoos. We collected 344 days of data and determined associations between recumbence and social, housing, management, and demographic factors. African elephants were recumbent less (2.1 hours/day, S.D. = 1.1) than Asian elephants (3.2 hours/day, S.D. = 1.5; P < 0.001). Nearly one-third of elephants were non-recumbent on at least one night, suggesting this is a common behavior. Multi-variable regression models for each species showed that substrate, space, and social variables had the strongest associations with recumbence. In the African model, elephants who spent any amount of time housed on all-hard substrate were recumbent 0.6 hours less per day than those who were never on all-hard substrate, and elephants who experienced an additional acre of outdoor space at night increased their recumbence by 0.48 hours per day. In the Asian model, elephants who spent any amount of time housed on all-soft substrate were recumbent 1.1 hours more per day more than those who were never on all-soft substrate, and elephants who spent any amount of time housed alone were recumbent 0.77 hours more per day than elephants who were never housed alone. Our results draw attention to the significant interspecific difference in the amount of recumbent rest and in the factors affecting recumbence; however, in both species, the influence of flooring substrate is notably important to recumbent rest, and by extension, zoo elephant welfare.

## Introduction

Obtaining adequate rest is essential for the good health and welfare of animals [1–2], yet few studies of zoo animal welfare focus on resting behaviors, perhaps due to the difficulty of measuring and interpreting these behaviors. For example, many species perform rest both while standing and recumbent; of these, the welfare implications of recumbent rest are better understood due to extensive research on cattle. Cattle are highly motivated to lie down [3], and cattle that have been deprived of opportunities for recumbence, feeding, and social contact will prioritize compensatory recumbence over other behaviors [4–5]. Reducing opportunities for cattle to lie down can also affect growth hormone levels [6] and result in various behavioral or physiological indications of stress [7]. In addition, cattle that spend more time standing are at a greater risk for lameness and hoof problems [8].

An important component of resting is sleep. Many species (e.g., cattle, horses, elephants; [2,9]) require recumbence for some types of sleep; in these species a lack of recumbence may lead to sleep deprivation. The health and welfare consequences of sleep deprivation have been well-studied in humans and laboratory animals. In a variety of mammalian species, sleep deprivation causes disruptions in vital biological processes including immune function [10–11], thermoregulation, energy conservation, tissue restoration, and higher cognitive function [12–16].

Like cattle and horses, the resting postures of African savanna elephants (*Loxodonta africana*) and Asian elephants (*Elephas maximus*) include both standing rest and recumbent rest. Standing rest often precedes recumbence [17], and recumbent elephants seem to quickly fall asleep: they are immediately motionless with their eyes closed [17], and display heavy, sometimes irregular respiration [18], twitching of the musculature and eyelids [17–18], snoring [19–20], and sometimes loud vocalizations [17]. Elephant sleep has not been described using EEG, so the exact nature of sleep occurring during recumbence is unknown. Regardless, recumbence is a natural resting behavior exhibited by elephants both in the wild [19–24] and in managed care [17–18,25–32].

In order to better understand recumbence in zoo elephants, we sought to quantify and describe recumbence, including its timing, patterns, and prevalence. We also sought to determine the potential associations between a variety of social, housing, management, and demographic factors and recumbence. As part of this process we tested three specific hypotheses. First, we hypothesized that zoo elephants who spent more time on hard substrates would be recumbent less. This intuitive relationship has been seen in cattle, where concrete floors result in significantly less time spent lying down [33]. If zoo elephants on concrete are experiencing a similar reduction in recumbence, they may be experiencing sleep deprivation or sleep disturbance, or be experiencing stress or frustration because of a reluctance to exhibit natural resting postures. Next, we hypothesized that the amount of space experienced would be positively correlated with recumbence. A relationship between space and recumbence is supported by research in cattle and horses, which has shown that recumbence increases when more stall space is provided [34–35]. Finally, we hypothesized that recumbence would decline with age based on previous research showing that adult elephants are recumbent less than infants, juveniles, and sub-adults [17,28,30,32,36].

Our study is the first large-scale investigation of African and Asian zoo elephant recumbence and was a component of the *Using Science to Understand Zoo Elephant Welfare* project, a multi-institutional collaborative effort to produce scientific data that will support decision making with regard to best practices in elephant management [37].

## Methods

### Ethics statement

This study was authorized by the management at each participating zoo and, where applicable, was reviewed and approved by zoo research committees. In addition, the study protocol was reviewed and approved by the Zoological Society of San Diego Institutional Animal Care and Use Committee N.I.H. Assurance A3675-01; Protocol 11–203. The study was non-invasive.

### Subjects and facilities

Zoos that were accredited members of the Association of Zoos and Aquariums in 2012 were eligible for participation in this study provided that they managed only African savanna or Asian elephants in a non-mixed species herd, and their herd included at least two adult female elephants who were not pregnant or experiencing severe illness or injury. A total of 49 zoos participated in the study. We used simplified random sampling to select two adult females (age ≥ 12 years) as subjects from each zoo; however, 26 zoos only had two eligible subjects so there was no randomization. In one case there were four subjects from one zoo; this zoo housed African and Asian elephants in separate exhibits. Three subjects were removed from the dataset prior to analysis because they were transferred between zoos or died during the 2012 study year.

### Data collection

All data were collected between May 2012 and November 2012. We used historical weather data [38] to select a one month data collection period at each location that minimized inter-zoo variation in predicted daily maximum temperature (range: 22.3 C to 34.1 C). We instructed zoos to collect five non-consecutive days of data (24 hours/day) from each subject within a one-month timeframe. Zoos could collect data from both subjects on the same day, or use an alternating schedule.

Leather anklets (Excelsior Leather, California, USA) [39–40] were custom-fit to elephants using measurements provided by participating zoos. The ends of the anklets had D-rings to which shackles and brummel hooks were attached. This hardware was used to secure the anklet in place without causing constriction. A pouch attached to each anklet contained a waterproof case (OtterBox Drybox OTR3-1000S, OtterBox, Colorado, USA) inside of which was a GPS data logger (used to collect data for a related study [40]) and an HOBO Pendant G Data Logger accelerometer (model UA-004-64, Onset Computer Corporation, Massachusetts, USA). Accelerometers are data loggers that can measure g-force and degree of tilt; we chose to evaluate recumbence using g-force measurement following previous studies of cattle recumbence using the same device [41–42]. The accelerometer was placed inside the anklet such that the x-axis was perpendicular to the ground pointing dorsally, and the y- and z-axes were parallel to the ground. We programmed the accelerometers to collect x-axis data at one-minute intervals. The total weight of the unit was approximately 1.2 kg depending on the anklet size and number of shackles used. We shipped the anklets to the zoos and elephant care staff attached the anklets to one of the front legs of each subject.

### Data processing

Of the 49 original participating zoos, 40 zoos successfully collected data from 72 elephants. We downloaded the data using HOBOware Pro software (v. 3.2.0, Onset Computer Corporation) and exported it into Microsoft Excel (Microsoft Corporation, Washington, USA). We then followed established data processing methods [42] by adding a constant (3.2) to all g-force values (range: -3.2 to 3.2) to make them positive (range: 0 to 6.4), then coding values < 2.55 as standing and ≥ 2.55 as lying. All lying values indicate accelerometer tilt of ≥ 50°, a cutoff selected based on visual observations of recumbence in cattle [42]. Before the study began we validated these methods for elephants by outfitting two subjects with anklets and videotaping their behaviors over two nights (data in S1 Appendix).

We omitted standing and lying bouts that consisted of only a single reading (e.g., a one-minute interval of “standing” sandwiched between “lying” bouts) because these readings may represent subtle leg movements during a period of consistent orientation [41,43]. We summed all other lying time to calculate recumbence (hours) for each day of data, and we averaged these daily values to calculate mean daily recumbence (hours/day). In addition, we calculated the nighttime (20:00–07:00) mean bout frequency by averaging the number of nightly recumbence bouts for each elephant, then averaging across all elephants. Finally, we calculated the nighttime mean bout duration by averaging the duration of nightly recumbent bouts for each elephant, then averaging across all elephants; however, we excluded nights on which elephants did not lay down to avoid under-estimating bout duration.

### Independent variables

Independent variables were selected based on hypotheses regarding their potential association with recumbence. Definitions for the variables selected for testing in this study are described in Table 1. Details on the collection and calculation of independent variables are presented in [40] and [44–47].

**Table 1:** Definitions of independent variables tested for correlation with mean daily recumbence.

Variable	Unit of Analysis	Unit	Time Scale	Description	Ref
Age	Elephant			Age of elephant (years)	[47]
Species	Elephant			African or Asian	[47]
Origin	Elephant			Captive or wild born	[47]
Space Experience				The average weighted (by percent time) size of all environments in which an elephant spent time	[44]
Total	Elephant	500 ft^2^	Overall, Day, Night	For all environment types	[44]
Indoor	Elephant	500 ft^2^	Overall, Day, Night	For indoor environments only	[44]
In/Out Choice	Elephant	500 ft^2^	Overall, Day, Night	For environments where there is a choice of indoors or outdoors	[44]
Outdoor	Elephant	500 ft^2^	Overall, Day, Night	For outdoor environments only	[44]
Space Experience per Elephant	Elephant	500 ft^2^	Overall, Day, Night	The area of all environments in which an elephant spent time, divided by the number of elephants sharing each environment, weighted by the percent time spent in each environment and averaged.	[44]
Percent Time				Sum of monthly percent time spent in category, averaged over time period	
Indoor	Elephant	%	Overall, Day, Night	Time spent in indoor environments	[44]
In/Out Choice	Elephant	%	Overall, Day, Night	Time spent in environments with an indoor/outdoor choice	[44]
Outdoor	Elephant	%	Overall, Day, Night	Time spent in outdoor environments	[44]
Soft Substrate	Elephant	%	Overall, Day, Night	Time spent in environment with 100% grass, sand, or rubber substrate	[44]
Hard Substrate	Elephant	%	Overall, Day, Night	Time spent in environment with 100% concrete or stone aggregate substrate	[44]
Housed Separately	Elephant	%	Overall, Day, Night	Time spent housed in a social group of one	[44]
Juveniles (<7 years old)	Elephant	%	Overall, Day, Night	Time spent in social groups where an elephant 7 years or younger was present	[44]
Social Experience	Elephant		Overall, Day, Night	The average weighted (by percent time) size of all social groups in which an elephant spent time	[44]
Animal Contact	Elephant		Overall, Day, Night	Max number of unique elephants focal animal is in contact with	[44]
Mean Daily Walking Distance	Elephant			Distance walked per day while outdoors, averaged over all days of data collection	[40]
Herd Size	Zoo			Total number of elephants at zoo	[44]
Temperature	Zoo			Average daily temperature at zoo, averaged over all days of data collection	[38]
Enrichment Program	Zoo			Standardized factor score created using a polychoric PCA to examine the frequency of use of the different components of an enrichment program	[45]
Enrichment Diversity	Zoo			Shannon diversity index score of enrichment activities types and frequencies conducted at zoo	[45]
Exercise Diversity	Zoo			Shannon diversity index score of exercise types and frequencies conducted at zoo	[45]
Foot Health	Zoo			Score of 0–12 indicating abnormalities on nails, pads, and interdigital space on any foot	[46]

A novel variable called Space Experience warrants further attention. Space Experience was based on data from detailed facility surveys [44] and was calculated by first taking the size of each environment in which an elephant spent time, and then multiplying it by the percentage of time the elephant spent in that environment. These weighted environment sizes were then averaged to calculate a representative value for each elephant [for additional details, see [44]]. This allows us to account for the complex housing conditions of zoo elephants, in which they may be shifted between environments of different sizes for varying amounts of time, including at night.

We also created novel environment type and flooring substrate variables. We first defined each space in which elephants spent time as indoors, outdoors or mixed based on detailed facility surveys [44]. Mixed environments were areas where elephants had a choice to move freely between indoor and outdoor spaces. We then defined multiple classes of flooring substrate: grass, sand, rubber padding, stone aggregate, concrete and categorized the types of substrates into hard surface (concrete and stone aggregate) and soft surface (grass, sand, and rubber padding), and determined the percent coverage for each substrate type for each environment. We wanted to calculate the time that elephants spent in contact with each substrate type so to confirm this we determined which environments were comprised of 100% hard and 100% soft substrate and calculated the percent time each elephant spent in environments that met these criteria from detailed housing time budgets [44].

We checked all continuous independent variables for outliers and removed any values that were greater than three standard deviations away from the mean. We adjusted some variables from continuous to binary because of zero-values for a high number of subjects. Adjusted variables included two space variables (Space Experience In/Out Choice and Percent Time In/Out Choice), two flooring variables (Percent Time Hard Substrate and Percent Time Soft Substrate) and two social variables (Percent Time Housed Separately and Percent Time Juveniles). The Space Experience variables were adjusted to a value of “per 500 ft^2^” to aid in the interpretation of Beta values.

### Statistical analysis

To determine whether there were interspecific differences in patterns of recumbence, we used two-sample Student’s t-tests assuming equal variances to test three null hypotheses: that both species had the same (1) mean daily recumbence; (2) mean number of nighttime recumbence bouts; and (3) mean duration of nighttime recumbence bouts. Upon discovering that many elephants were highly non-recumbent, or intermittently non-recumbent, we used Fisher's Exact Test to determine if the proportion of elephants displaying non-recumbence differed by species.

The regression models were fitted using generalized estimating equations (GEE), which allow for the individual elephant to be used as the unit of analysis, account for clustering of individuals within zoos, and support repeated measurement [48–49]. Zoos were treated as random effects and an independent correlation structure was specified [50]. Multi-variable regression models were built by first assessing individual predictors at the univariate level. During this univariate analysis we found a significant correlation between species and mean daily recumbence (β = 1.073, df = 71, P < 0.001), thus, we created separate models for African elephants and Asian elephants. We assessed individual predictors at the univariate level and then at the bivariate level with demographic variables (age and origin). Based on the fact that age and origin were likely to have an effect on both outcome and the tested input variable, these variables were tested as potential confounding variables [51–52]. Confounding variables (those that altered the beta values of input variables by more than 10% during bivariate analysis) were included in all models, and any variables correlated with recumbence (P < 0.15) following the univariate and bivariate assessments were retained for evaluation in the hierarchical model building process. The hierarchical selection was based on quasi-likelihood under the independence model criterion (QIC) values and parameter estimates of explanatory variables.

Models exhibiting multi-collinearity, as defined by a variance inflation factor (VIF) of greater than 10 and a Condition Index (CI) of greater than 30, were not considered for further analysis. The African elephant model used an autoregressive correlation matrix type, while the Asian elephant model specified an independent correlation structure. Statistical analyses were conducted by using SAS software, version 9.3 [PROC GENMOD, with options REPEATED, CORR = IND or AR, and DIST = NORMAL; SAS Institute, Inc., Cary, NC].

## Results

### Summary of recumbence data

**Fig 1:**
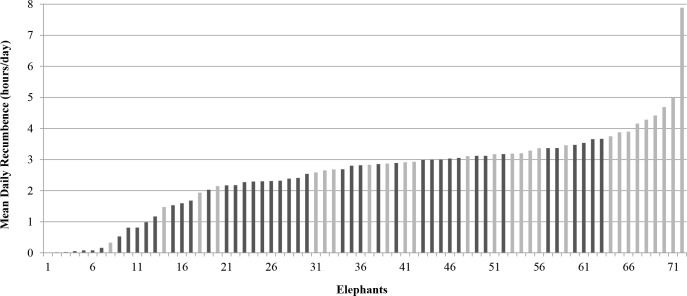
Mean daily recumbence in zoo elephants. Black bars indicate African (n = 44), grey bars indicate Asian (n = 28).

Our final dataset included 344 days of data, collected between May 7, 2012 and November 1, 2012, from 72 elephants at 40 zoos. A full 24 hours of data were collected on 277 days; on 67 days anklets were removed before a full 24 hours of data were collected, resulting in an average of 31 minutes (range: 2 to 105) when recumbence data were not available. Any recumbence occurring during these times was not recorded, thus, the mean daily recumbence values may slightly underestimate actual values. For the majority of elephants (61/72) five days of data were collected, but in some cases the data were limited to four days (6/72) or three days (5/72). The 72 elephants included 44 African elephants (61.1%) and 28 Asian elephants (39.9%) (Fig 1). The mean age of African elephants was 32.6 years (range: 20 to 52); the mean age of Asian elephants was 40.0 years (range: 16 to 61).

**Fig 2:**
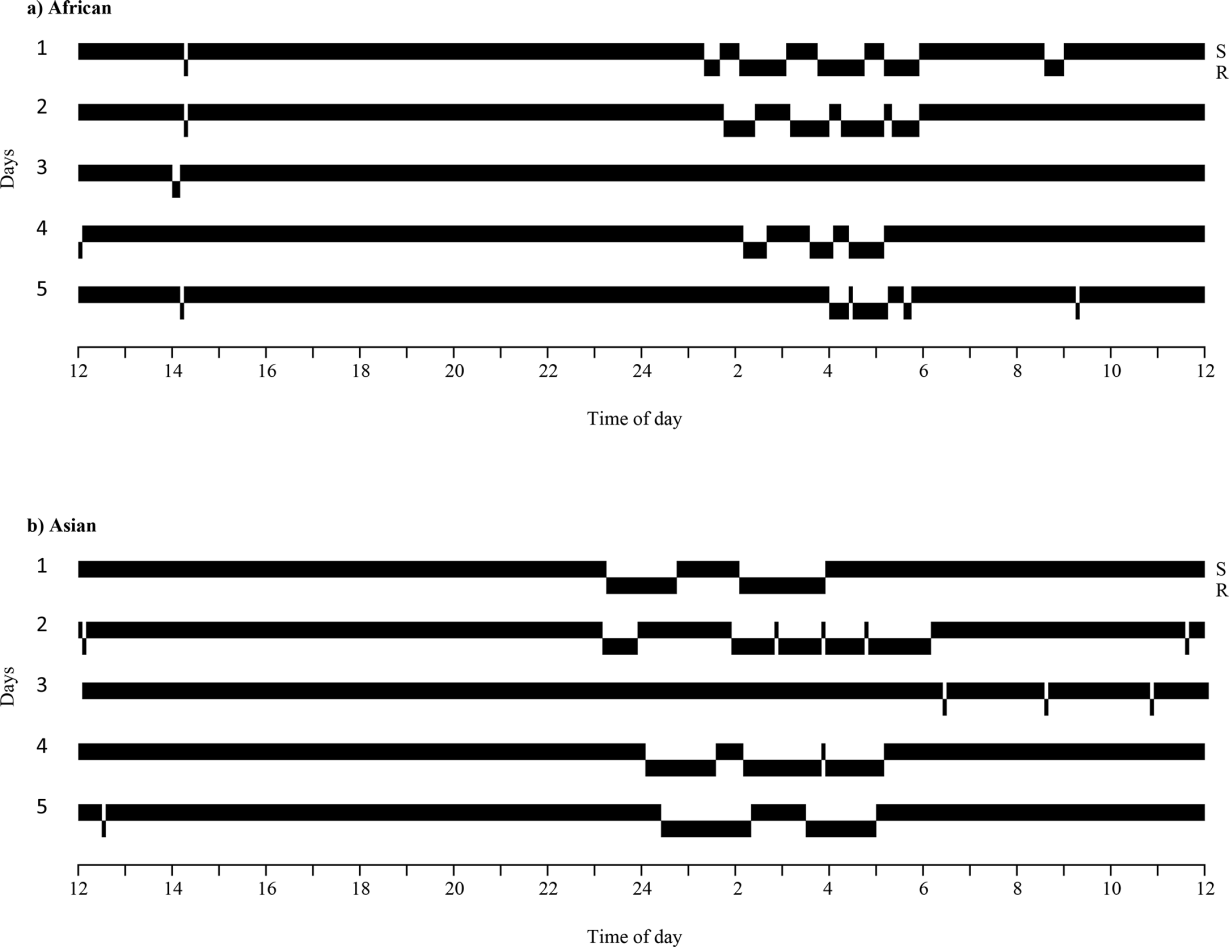
**Standing and recumbence patterns of a representative African (a) and Asian (b) zoo elephant over five days.** These elephants were coincidentally both non-recumbent on the third day of data collection. S = standing; R = recumbent.

**Fig 3:**
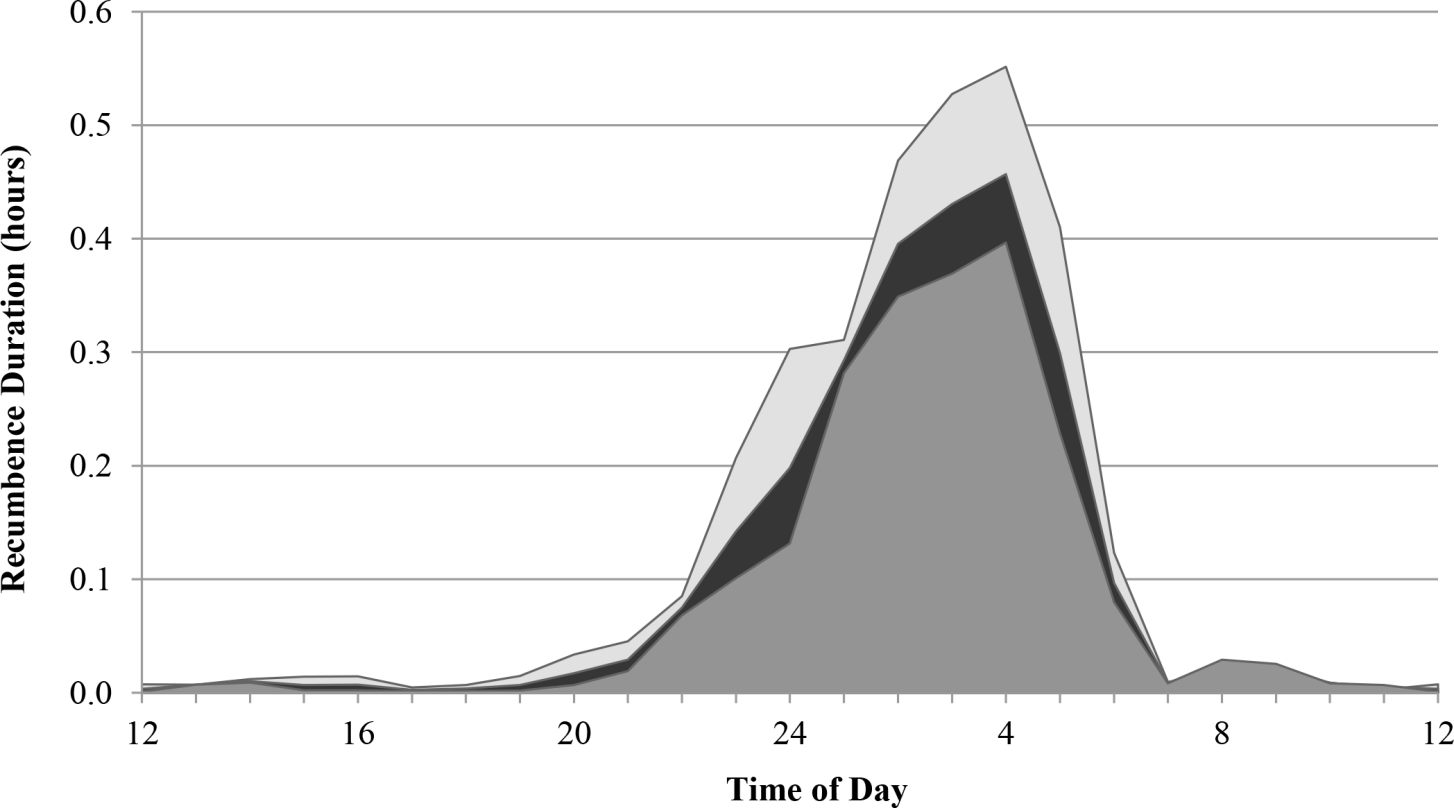
Recumbence profile showing daily distributions of recumbence in zoo elephants. Light grey indicates Asian elephants (n = 28), dark grey indicates African elephants (n = 44), black shows both species combined (n = 72). The lines of the curves connect mean hourly values. Areas under the curves represent total time recumbent.

Mean daily recumbence for all elephants was an average of 2.6 hours/day; the individual mean recumbence value and standard error for all subjects is available in S2 Appendix. African elephants had significantly lower recumbence (2.1 hours/day) than Asian elephants (3.2 hours/day) (t(70) = -3.48, P < 0.001, two-tailed) (Table 2). Mean nighttime bout frequencies in African and Asian elephants were not significantly different at 3.1 bouts/night for both species (t(70) = 0.05, P = 0.96, two-tailed) (Table 2). Africans and Asians were different in mean nighttime bout duration (t(70) = -4.95, P < 0.001) with African elephants recumbent an average of 39 minutes/bout and Asian elephants recumbent an average of 66 minutes/bout (Table 2). Variations in the standing and lying patterns of African and Asian elephants can be seen by comparing the behavior of representative individuals (Fig 2). African and Asian elephants showed similar mean daily recumbence profiles: recumbence rarely occurred during the day, started to increase at 20:00, and reached a peak at 04:00 before sharply dropping off (Fig 3).

**Table 2:** Summary of recumbence data for African and Asian zoo elephants. A t-test was used to test for a difference in the means between species in each of the variables (*P < 0.05).

		Combined (n = 72)	African (n = 44)	Asian (n = 28)	P	
Mean Daily Recumbence (Hours/Day)	Mean	2.6	2.1	3.2	<0.01	*
	Min.	0.0	0.0	0.0		
	Max.	7.9	3.7	7.9		
	S.D.	1.4	1.1	1.5		
Mean Nighttime Bout Frequency (Bouts/Night)	Mean	3.1	3.1	3.1	0.96	
	Min.	0.0	0.0	0.0		
	Max.	6.3	5.8	6.3		
	S.D.	1.4	1.5	1.4		
Mean Nighttime Bout Duration (Hours/Bout)	Mean	0.8	0.7	1.1	<0.001	*
	Min.	0.0	0.0	0.0		
	Max.	2.0	1.4	2.0		
	S.D.	0.4	0.3	0.4		

A number of elephants showed some form of non-recumbence. Seven elephants (six African, one Asian) were classified as highly non-recumbent because they lay down for less than one hour total over five days of data collection. An additional 15 elephants (nine African, six Asian) were classified as intermittently non-recumbent because they lay down for less than ten minutes per day on three days of data collection (n = 1), two days (n = 1), or one day (n = 13). There was no significant difference in the number of highly non-recumbent elephants by species (Fisher's Exact Test, P = 0.252) or the number of intermittently non-recumbent elephants by species (Fisher's Exact Test, P = 1.00).

### Univariate analyses

We evaluated a variety of demographic, housing, social, and management factors for association with recumbence (Tables 3 and 4). In the African elephant univariate tests (Table 3), mean daily recumbence was negatively correlated with Age and Percent Time Hard Substrate Overall, Day, and Night. Recumbence was also negatively correlated with Social Experience Day, and Percent Time Housed Separately Overall and at Night. There was a positive correlation between recumbence and the variables Space Experience Total Night, Space Experience Outdoor Night, and Space Experience per Elephant Night. We were unable to test for a correlation between recumbence and Origin due to the small number of African elephants who had been imported from a range country (1/44).

**Table 3:** Univariate correlations between mean daily recumbence and independent variables in African zoo elephants.

			Overall			Day ^a^			Night^a^		
Variables	+/- ^b^	Reference	n	Beta	P-value	n	Beta	P-value	n	Beta	P-value
*Demographics*											
Age	-		44	-0.087	<0.001*						
*Space*											
Space Experience Total (500 ft^2^)	+		42	0.003	0.363	44	-0.000	0.925	42	0.007	<0.001*
Space Experience Indoor (500 ft^2^)	+		43	-0.069	0.259	44	-0.046	0.414	43	-0.063	0.321
Space Experience Outdoor (500 ft^2^)	+		43	0.001	0.830	44	-0.001	0.663	42	0.007	0.002*
Space Experience In/Out Choice (500 ft^2^)		ref = 0%	18			22			18		
	+	>0%	26	0.137	0.681	22	-0.945	0.749	26	0.090	0.782
Space Experience per Elephant	+		44	0.005	0.379	44	-0.003	0.631	42	0.028	0.013*
Percent Time Indoor	-		44	-0.006	0.456	44	0.001	0.944	44	-0.007	0.191
Percent Time Outdoor	+		44	0.000	0.968	44	-0.002	0.717	44	0.002	0.750
Percent Time In/Out Choice		ref = 0%	18			22			18		
	+	>0%	26	0.137	0.681	22	-0.150	0.622	26	0.137	0.681
*Flooring*											
Percent Time Hard Substrate		ref = 0%	20			24			21		
	-	>0%	24	-0.673	0.018*	20	-0.647	0.029*	23	-0.749	0.007*
Percent Time Soft Substrate		ref = 0%	22			26			25		
	+	>0%	22	0.040	0.901	18	0.057	0.860	19	0.146	0.667
*Social*											
Herd Size	+		44	0.022	0.711						
Animal Contact	+		42	-0.136	0.192	42	-0.136	0.192	42	-0.055	0.767
Social Experience	+		42	-0.297	0.221	42	-0.192	0.146^	42	0.009	0.974
Percent Time Juveniles (age <7)		ref = 0%	33			33			34		
	+	>0%	11	0.017	0.963	11	0.017	0.963	10	-0.094	0.823
Percent Time Housed Separately		ref = 0%	28			33			28		
	-	>0%	15	-0.601	0.098^	11	-0.217	0.580	16	-0.704	0.054^
*Management*											
Enrichment Diversity	+		42	-0.833	0.269						
Enrichment Program	+		42	-0.084	0.652						
Exercise Diversity	+		42	0.103	0.740						
*Other*											
Foot Health	-		39	-0.046	0.614						
Temperature	+		44	-0.021	0.268						
Walking Distance	+		32	0.023	0.829						

^*P* value <0.15 utilized as threshold significant level for model building

*P value <0.05

^a^ Day and night are defined as the number of hours in a 24 hour period considered daytime or nighttime according to management schedule.

^b^ Hypothesized direction of relationship between mean daily recumbence and variable.

**Table 4:** Univariate correlations between mean daily recumbence and independent variables in Asian zoo elephants.

			Overall			Day ^a^			Night ^a^		
Variables	+/- ^b^	Reference	n	Beta	P-value	n	Beta	P-value	n	Beta	P-value
*Demographics*											
Age	-		28	0.016	0.236						
Origin		ref^c^ = Wild	21								
	+	Captive	7	-0.291	0.576						
*Space*											
Space Experience Total (500 ft^2^)	+		27	0.008	0.462	28	-0.006	0.494	27	0.009	0.465
Space Experience Indoor (500 ft^2^)	+		27	-0.081	0.553	28	-0.136	0.028*	28	-0.103	0.267
Space Experience Outdoor (500 ft^2^)	+		27	0.003	0.647	27	0.002	0.718	27	0.001	0.910
Space Experience In/Out Choice (500 ft^2^)		ref = 0%	11			19			13		
	+	>0%	17	0.399	0.511	9	0.211	0.633	15	0.213	0.695
Space Experience per Elephant	+		28	0.038	0.314	28	0.021	0.435	28	0.026	0.403
Percent Time Indoor	-		27	-0.012	0.432	27	-0.029	0.038*	28	-0.011	0.261
Percent Time Outdoor	+		28	0.008	0.397	28	0.004	0.647	28	0.006	0.444
Percent Time In/Out Choice		ref = 0%	11			19			13		
	+	>0%	17	0.399	0.511	9	0.211	0.633	15	0.213	0.695
*Flooring*											
Percent Time Hard Substrate		ref = 0%	10			11			13		
	-	>0%	17	-0.049	0.919	16	0.151	0.770	15	-0.815	0.079^
Percent Time Soft Substrate		ref = 0%	11			13			16		
	+	>0%	17	0.971	0.029*	15	0.858	0.064^	12	0.793	0.123^
*Social*											
Herd Size	+		26	-0.223	0.447						
Animal Contact	+		27	-0.342	0.323	27	-0.342	0.323	27	-0.685	0.099^
Social Experience	+		28	-0.760	0.130^	28	-0.659	0.183	28	-0.574	0.212
Percent Time Juveniles (age <7)		ref = 0%	24			24			24		
	+	>0%	4	-0.348	0.614	3	-0.761	0.150	3	0.136	0.775
Percent Time Housed Separately		ref = 0%	9			15			14		
	-	>0%	19	1.051	0.029*	13	0.998	0.050^	14	0.936	0.045*
*Management*											
Enrichment Diversity	+		23	0.585	0.730						
Enrichment Program	+		23	-0.052	0.751						
Exercise Diversity	+		22	-0.220	0.773						
*Other*											
Foot Health	-		24	0.050	0.634						
Temperature	+		28	0.026	0.554						
Walking Distance	+		23	-0.079	0.418						

^*P* value <0.15 utilized as threshold significant level for model building

*P value <0.05

^a^ Day and night are defined as the number of hours in a 24 hour period considered daytime or nighttime according to management schedule.

^b^ Hypothesized direction of relationship between mean daily recumbence and variable.

^c^ The reference value (ref =) was the baseline value used when calculating univariate correlations with these binary variables.

In the Asian elephant univariate tests (Table 4), mean daily recumbence was negatively correlated with Space Experience Indoor Day and Percent Time Indoor Day, as well as Percent Time Hard Substrate Night, Animal Contact Night, and Social Experience Overall. Recumbence was positively correlated with Percent Time Soft Substrate Overall, Day, and Night, and Percent Time Housed Separately Overall, Day, and Night.

The population level descriptive statistics for the variables that were significant in African and Asian elephant univariate analyses are shown in Table 5.

**Table 5:** Descriptive statistics for independent variables included in the multi-variable modeling process. The sample size and mean age of elephants used in the correlation is provided.

						Variable				
Species	Variable	Mgmt.	Reference	n	Mean Age	Mean	SD	Min	Max	Median
African	Age			44	-	32.6	6.6	20	52	32
African	Space Experience Total (500 ft^2^)	Night		42	33	44.0	51.1	0. 9	227.4	27.5
African	Space Experience Outdoor (500 ft^2^)	Night		43	33	57.3	59.7	0	244.0	37. 9
African	Space Experience per Elephant (500 ft^2^)	Night		42	33	14.2	11.5	0.8	45.0	5.9
African	Percent Time Hard Substrate	Overall	ref = 0%	20	31	-	-	-	-	-
			>0%	24	35	15.8	8.5	2.3	32.2	15.0
African	Percent Time Hard Substrate	Day	ref = 0%	24	31	-	-	-	-	-
			>0%	20	35	10.5	7.8	1.1	24.2	11.3
African	Percent Time Hard Substrate	Night	ref = 0%	21	31	-	-	-	-	-
			>0%	23	34	25.5	16.7	5.6	53.3	23.5
African	Social Experience	Day		42	34	19.9	13.7	6.8	56.3	14.7
African	Percent Time Housed Separately	Overall	ref = 0%	28	31	-	-	-	-	-
			>0%	15	35	22.9	17.7	1.4	57.1	19.9
African	Percent Time Housed Separately	Night	ref = 0%	28	31	-	-	-	-	-
			>0%	16	35	44.5	34.1	3.8	100. 0	37.6
Asian	Space Experience Indoor (500 ft^2^)	Day		28	40	1.7	2.2	0	8.0	1.1
Asian	Percent Time Indoor	Day		27	40	12.3	11.8	0	36.3	10
Asian	Percent Time Hard Substrate	Night	ref = 0%	13	39	-	-	-	-	-
			>0%	15	41	20.2	19.3	2.2	53.4	8.6
Asian	Percent Time Soft Substrate	Overall	ref = 0%	11	44	-	-	-	-	-
			>0%	17	37	15.8	11.1	0.4	31.4	18.4
Asian	Percent Time Soft Substrate	Day	ref = 0%	13	46	-	-	-	-	-
			>0%	15	35	10.4	9.5	0.4	29.2	8.6
Asian	Percent Time Soft Substrate	Night	ref = 0%	16	42	-	-	-	-	-
			>0%	12	37	27.0	9.3	14.7	48.5	26.3
Asian	Animal Contact	Night		27	41	1.0	0.7	0	3.0	1. 0
Asian	Social Experience	Overall		28	40	18.9	10.3	0.7	45	15.7
Asian	Percent Time Housed Separately	Overall	ref = 0%	9	41	-	-	-	-	-
			>0%	19	40	38.7	38.2	1.8	100	25
Asian	Percent Time Housed Separately	Day	ref = 0%	15	42	-	-	-	-	-
			>0%	13	37	39.2	39.8	5.3	100	19.5
Asian	Percent Time Housed Separately	Night	ref = 0%	14	35	-	-	-	-	-
			>0%	14	45	68.5	37.8	8.1	100	86.1

### African elephant multi-variable model

The African elephant multi-variable model (Table 6) includes Beta estimates for Percent Time Hard Substrate Overall and Space Experience Outdoor Night. Beta estimates reflect the magnitude of the effect of the independent variables on recumbence as described below, but it is important to note that this effect is conditional on the effects of the other independent variables in each model. The model demonstrates an association between flooring and recumbence such that elephants who spent any time on 100% concrete or stone aggregate substrate (“all-hard”) were recumbent 0.6 hours less per day than elephants who spent no time on all-hard substrate. The model also demonstrates a positive relationship between the amounts of outdoor space an elephant experiences at night and recumbence: elephants who experienced an additional 500 ft^2^ of outdoor space during the night were recumbent 0.006 hours more per day; this translates to a 0.5 hour increase per additional acre.

**Table 6:** African elephant mean daily recumbence multi-variable model (*P < 0.05)^1^.

Variable	Beta Estimate	Standard Error	Pr > |Z|
Intercept	2.194	0.217	<0.001
0% time hard substrate	-	-	-
>0% time hard substrate	-0.600	0.245	0.014*
Space experience outdoor night	0.006	0.002	0.001*

^1^ Variance Inflation Factor = 1.051; Maximum Condition Index = 3.755

### Asian elephant multi-variable model

The Asian elephant multi-variable model (Table 7) includes Beta estimates for Percent Time Soft Substrate Overall and Percent Time Housed Separately Night. The model shows that elephants who spent any time on 100% grass, sand, or rubber substrate (“all-soft”) were recumbent 1.1 hours more per day than elephants who spent no time on all-soft substrate. The variable Age is included as a confounder of Percent Time Soft Substrate Overall indicating that age is a factor that is both related to being on hard surfaces and being recumbent; age is included in the model to control for its potential effects. The model also demonstrates a positive relationship between whether an elephant is housed alone at night and recumbence, such that elephants who were alone for any amount of time during the night were recumbent 0.77 hours more per day than elephants who were never housed alone during the night.

**Table 7:** Asian elephant mean daily recumbence multi-variable model (*P < 0.05)^1^.

Variable	Beta Estimate	Standard Error	Pr > |Z|
Intercept	1.646	0.733	0.025
0% time soft substrate	-	-	-
>0% time soft substrate	1.056	0.352	0.003*
0% time housed separately	-	-	-
>0% time housed separately	0.770	0.387	0.047*
Age	0.016	0.107	0.358

^1^ Variance Inflation Factor = 1.005; Maximum Condition Index = 4.112

## Discussion

### Recumbence patterns, timing and prevalence

We found that on average, African elephants lay down for just over two hours per day and Asian elephants lay down for just over three hours per day. Recumbence occurred almost exclusively at night. Our results correspond with other large studies of recumbence in elephants under managed care: adult female African elephants (n = 11) in European zoos lay down an average of 2.0 hours per night [32], while adult female Asian elephants (n = 8) at a zoo and circus lay down an average of 3.4 hours per night [17]. Our results also correspond with available data from wild African elephants (n = 4) that lay down for between one and two hours per night on average [19]; no data are available for nighttime recumbence in wild Asian elephants.

Sleep appears to be the primary function of recumbence, as elephants entering a recumbent posture appear to fall asleep almost immediately [17]. Thus, differences in recumbence between African and Asian elephants likely reflect interspecific variation in sleep requirements. Sleep patterns appear to be determined primarily by ecological variables [53]. For example, some species show a trade-off between time available for sleep and time available for foraging [54]. Wild Asian elephants may inhabit more resource-rich areas than wild African elephants, allowing them to fulfill their nutritional requirements in less time. Asian zoo elephants spend significantly less time feeding than African zoo elephants [45], but whether this is attributable to the species’ natural history or a difference in zoo feeding methods is not known. Another ecological variable that may explain interspecific variation in sleep is predation risk. Species that sleep more tend to use less exposed sleeping sites [54–55]. Asian elephants may be more likely than African (savanna) elephants to inhabit dense forested areas that conceal them from predators and allow for more sleep. Regardless of the cause of the difference in recumbence, our results suggest that animal welfare indices based on behavior should take into account the potential for significant differences between elephant species.

African and Asian elephants showed similar timing of recumbence behavior, being mainly recumbent between 01:00 and 05:00 with a peak at 04:00, in agreement with other studies [17, 19]. The timing of recumbence may have management implications. For example, zoos that have nighttime elephant care staff or automated feeders should plan management routines so as to minimize disturbances to sleeping elephants during peak recumbence hours; indeed, automated feeders have been shown to interrupt recumbence in zoo elephants [32].

### Non-recumbence

We observed some form of non-recumbence in nearly one-third of elephants in our study. Age-related health problems (e.g., arthritis) could be limiting recumbence in some individuals [29], and although a related study [46] provided musculoskeletal health data for some of our subjects we were unable to test for an association with recumbence due to limited variability in joint health in the subjects. We also observed the behavior across a range of ages, so health problems are unlikely to be the sole cause of non-recumbence. We considered that non-recumbence may be a normal and adaptive behavior in elephants. For example, animals living in groups may use vigilance to increase the probability of predator detection [56]. Although wild African elephants are rarely vigilant during the day [54], vigilance may be more important at night when the majority of predation attempts on elephants occur [57–58]. Non-recumbence may also be a specific form of vigilance called sentinel behavior. Zoo elephants have been observed standing in close proximity to a recumbent elephant for extended periods of time [30] and “taking turns” being recumbent [25]. However, no studies of zoo elephants have closely examined vigilance or sentinel behavior. Finally, non-recumbence may be an abnormal behavioral consequence of the zoo environment, with no physiological or ecological function, and may be caused by stress, disturbance, or some other unmeasured variable. In this case, the welfare of non-recumbent elephants may be impacted by sleep deprivation. Whether zoo elephants are able to make up for lost sleep the night after exhibiting non-recumbence could not be tested with our dataset because we collected data on non-consecutive nights, however, rebound recumbence is an area of possible future research.

### Substrate

Of all the independent variables we tested, substrate had the strongest association with recumbence, and a substrate variable was present in nearly every model during the model-building process. Our African model showed that elephants who spent time on all-hard substrate (concrete or stone aggregate) were recumbent 0.6 hours per day less than elephants who were never on all-hard substrate. Along the same lines, the Asian model showed an increase in recumbence of 1.1 hours per day for elephants who spent time on all-soft substrate (grass, sand, or rubber) when compared to elephants who were never on all-soft substrate.

Our results add to a growing body of evidence suggesting that hard substrate negatively impacts animal welfare. Concrete has been associated with higher rates of sole hemorrhages [59] and swollen knees [60] in cattle, and with incidents of foot and joint disease in zoo elephants [46]. Meanwhile, the reduction and removal of hard substrate from zoo elephant exhibits is already underway. A 2006 survey [61] following up on 1997 survey results [62] found that the proportion of concrete flooring in elephant barns had reduced 22% in the intervening years. In addition, nearly half of responding zoos planned to further reduce the proportion of concrete flooring in their indoor facilities over the next 10 years [61]. Despite these ongoing efforts, we found that 18 of 40 zoos in our study (45%) had elephants housed in environments with all-hard substrate at some time in 2012. This is in addition to the time these elephants spent in mixed substrate environments that included hard and soft substrate. The continued prevalence of hard substrate in zoo environments indicates that zoos must remain proactive in their attempts to incorporate soft substrate into both indoor and outdoor areas. Furthermore, we suggest continued research into soft substrate types (i.e., sand, grass, and rubber) in order to determine which are most effective at promoting health, welfare, and natural behaviors in zoo elephants. For example, research in horses has shown that despite straw and wood shavings both being arguably soft substrate, horses that are given a choice between the two preferentially spend time on straw [63] and exhibit more bedding-related activities [63] and lateral recumbence [64] on straw. Finally, our model suggests that substrate directly affects zoo elephant recumbence; that is, it is not a proxy for other related measurements such as time spent inside or outside.

### Space

We found a positive correlation between outdoor Space Experience at night and recumbence in African elephants. For example, an African elephant that experienced an additional acre of outdoor space at night increased their recumbence by 0.48 hours in the final model–a potentially important contribution to the mean daily recumbence of an African elephant.

There are a variety of ways by which zoos can increase outdoor Space Experience at night. Providing access to a consistent amount of additional space is certainly one way. However, Space Experience allows for a flexible consideration of both space and time, so zoos can work within their own housing and management constraints to find ways to increase Space Experience by altering the amount of time elephants are housed in different exhibit configurations (for more details see [44]). Whether increases in Space Experience will eventually begin to reach a level of diminishing returns is an important area for future research.

### Age

We found a negative correlation between age and recumbence in our African (but not Asian) elephant model: as adult African elephants got older, they were recumbent less and less. Whether this trend is related to age-related health problems or merely reflects changes in sleep requirements (or both), we cannot say as research on senescence and sleep has not been conducted with elephants. However, many age-related changes occur in rodent sleep patterns, including a reduction in total sleep time and reduction in the duration of uninterrupted bouts of sleep (i.e. sleep fragmentation) with increased age [65], so future research into age-related sleep changes in elephants is warranted.

### Social

The initiation and termination of recumbence bouts is often synchronized amongst elephants in zoos [17, 26] and in the wild [19]. This suggests that recumbence is a highly social behavior. Although not one of our three focal hypotheses, we predicted that elephants who were never alone would show more recumbence, assuming that a more natural social environment would be more likely to result in the expression of natural behaviors. However, the Asian elephant model showed a positive correlation between recumbence and time housed separately. Why might Asian elephants who spend time alone be more recumbent? One possible explanation is that elephants housed alone do not experience overcrowding. Cattle, for example, were significantly less recumbent when the number of cows per stall increased by 50% [66]. However, we found no correlation between Asian elephant recumbence and social density, as measured by our Space Experience per Elephant variable. An alternate explanation is that being housed alone eliminates the possibility of physical disruption of rest by other members of a social group. In cattle, recumbence patters are related to social rank, and subordinate cattle are recumbent significantly less than middle-ranked or high-ranked cows [67], presumably because dominant cows physically disturb lower-ranking cows during resting periods. Future research will be needed to better understand the nocturnal social lives of elephants and their effect on rest and recumbence.

Our study used a standardized methodology to complete a large-scale investigation of African and Asian zoo elephant recumbence. This rare look into the resting behavior of zoo elephants resulted in a few notable conclusions. First, our finding that Asian elephants have significantly higher mean daily recumbence than African elephants suggests that animal welfare researchers should remain vigilant of possible species differences in zoo elephants when using behavioral indices as a tool to measure welfare. We also observed that nearly one-third of the elephants were non-recumbent on at least one night; this could indicate an important animal welfare concern and as such more research should be directed at determining the causes and effects of being non-recumbent. Finally, we established an association between recumbence and substrate, which supports continued efforts by zoos to replace hard substrate with soft substrate in order to provide zoo elephants with an environment where they can comfortably express recumbence behavior.

## Supporting Information

S1 Appendix Validation test of accelerometer using video analysis. Two Asian zoo elephants wore accelerometers in anklets for two consecutive nights and accelerometer data were compared with video recordings of recumbence activity. Additionally, Subject #2 wore a second accelerometer in the same anklet to test inter-accelerometer reliability. The most notable deviation between the accelerometer data and the video data occurs in bouts 2 and 3 of Subject #2, in which the accelerometers record two separate bouts during what the video records as one single, longer bout.
(DOCX)

S2 Appendix Mean recumbence and standard error for all subjects (n = 72). (DOCX)
