# Supplementary material for: Recumbence Behavior in Zoo Elephants: Determination of Patterns and Frequency of Recumbent Rest and Associated Environmental and Social Factors
Source: PLoS One. 2016 Jul 14;11(7):e0153301. doi: 10.1371/journal.pone.0153301 (PMC4945027; doi:10.1371/journal.pone.0153301)
Supplement: S1 Appendix — Two Asian zoo elephants wore accelerometers in anklets for two consecutive nights and accelerometer data were compared with video recordings of recumbence activity. Additionally, Subject #2 wore a second accelerometer in the same anklet to test inter-accelerometer reliability. The most notable deviation between the accelerometer data and the video data occurs in bouts 2 and 3 of Subject #2, in which the accelerometers record two separate bouts during what the video records as one single, longer bout. (DOCX) [file pone.0153301.s001.docx]

**S1 Appendix. Validation test of accelerometer using video analysis.** Two Asian zoo elephants wore accelerometers in anklets for two consecutive nights and accelerometer data were compared with video recordings of recumbence activity. Additionally, Subject #2 wore a second accelerometer in the same anklet to test inter-accelerometer reliability. The most notable deviation between the accelerometer data and the video data occurs in bouts 2 and 3 of Subject #2, in which the accelerometers record two separate bouts during what the video records as one single, longer bout.

| Subject #1^1^ | | |  | Subject #2^2^ | | | |
| --- | --- | --- | --- | --- | --- | --- | --- |
|  | Duration (h:mm) | |  |  | Duration (h:mm) | | |
| Bout | Accelerometer | Video |  | Bout | Accelerometer A | Accelerometer B | Video |
| 1 | 0:21 | 0:11 |  | 1 | 0:25 | 0:26 | 0:27 |
| 2 | 0:23 | 0:24 |  | 2 | 0:20 | 0:20 |  |
| 3 | 0:51 | 0:53 |  | 3 | 0:26 | 0:25 | 0:57 |
| 4 | 0:47 | 0:48 |  | 4 | 1:09 | 1:09 | 1:19 |
| 5 | 0:50 | 0:51 |  | 5 | 1:52 | 1:52 | 1:52 |
| Sum | 3:12 | 3:07 |  | 6 | 0:48 | 0:48 | 0:47 |
|  |  |  |  | 7 | 0:41 | 0:39 | 0:40 |
|  |  |  |  | 8 | 1:20 | 1:20 | 1:21 |
|  |  |  |  | 9 | 0:44 | 0:45 | 0:46 |
|  |  |  |  | 10 | 1:29 | 1:30 | 1:30 |
|  |  |  |  | Sum | 9:14 | 9:14 | 9:39 |

^1^Paired t-tests found no significant difference between the accelerometer and video data (t(4) = 0.44, P = 0.681, two-tailed).

^2^Paired t-tests found no significant difference between accelerometer A data and video data (t(8) = -1.46, P = 0.183, two-tailed), accelerometer B data and video data (t(8) = -1.41, P = 0.196, two-tailed), and accelerometer A data and accelerometer B data (t(9) = -0.00, P = 1.000, two-tailed).
