# Supplementary material for: Recumbence Behavior in Zoo Elephants: Determination of Patterns and Frequency of Recumbent Rest and Associated Environmental and Social Factors
Source: PLoS One. 2016 Jul 14;11(7):e0153301. doi: 10.1371/journal.pone.0153301 (PMC4945027; doi:10.1371/journal.pone.0153301)
Supplement: S2 Appendix — (DOCX) [file pone.0153301.s002.docx]

**S2 Appendix. Mean recumbence and standard error for all subjects (n = 72).**

| **Daily Recumbence** | | | | | | | | | | |
| --- | --- | --- | --- | --- | --- | --- | --- | --- | --- | --- |
| Subject | Mean | Standard Error |  | Subject | Mean | Standard Error |  | Subject | Mean | Standard Error |
| 1 | 0.0 | 0.0 |  | 25 | 2.3 | 0.2 |  | 49 | 3.1 | 0.5 |
| 2 | 0.0 | 0.0 |  | 26 | 2.3 | 0.6 |  | 50 | 3.1 | 0.2 |
| 3 | 0.0 | 0.0 |  | 27 | 2.3 | 0.6 |  | 51 | 3.1 | 0.5 |
| 4 | 0.1 | 0.0 |  | 28 | 2.4 | 0.2 |  | 52 | 3.2 | 0.4 |
| 5 | 0.1 | 0.0 |  | 29 | 2.4 | 0.7 |  | 53 | 3.2 | 0.6 |
| 6 | 0.1 | 0.0 |  | 30 | 2.5 | 0.8 |  | 54 | 3.2 | 0.8 |
| 7 | 0.2 | 0.0 |  | 31 | 2.5 | 0.3 |  | 55 | 3.3 | 0.4 |
| 8 | 0.3 | 0.1 |  | 32 | 2.6 | 0.5 |  | 56 | 3.3 | 0.3 |
| 9 | 0.5 | 0.4 |  | 33 | 2.7 | 0.5 |  | 57 | 3.4 | 0.5 |
| 10 | 0.8 | 0.3 |  | 34 | 2.7 | 0.3 |  | 58 | 3.4 | 0.5 |
| 11 | 0.8 | 0.3 |  | 35 | 2.8 | 0.4 |  | 59 | 3.5 | 1.0 |
| 12 | 1.0 | 0.1 |  | 36 | 2.8 | 0.3 |  | 60 | 3.5 | 0.4 |
| 13 | 1.2 | 0.3 |  | 37 | 2.8 | 0.3 |  | 61 | 3.5 | 0.3 |
| 14 | 1.5 | 0.5 |  | 38 | 2.8 | 0.4 |  | 62 | 3.6 | 0.4 |
| 15 | 1.5 | 0.4 |  | 39 | 2.9 | 0.9 |  | 63 | 3.7 | 0.2 |
| 16 | 1.6 | 0.7 |  | 40 | 2.9 | 0.5 |  | 64 | 3.8 | 1.1 |
| 17 | 1.7 | 0.8 |  | 41 | 2.9 | 1.0 |  | 65 | 3.8 | 0.4 |
| 18 | 1.9 | 0.5 |  | 42 | 2.9 | 0.7 |  | 66 | 3.9 | 1.1 |
| 19 | 2.0 | 0.8 |  | 43 | 2.9 | 0.6 |  | 67 | 4.2 | 1.2 |
| 20 | 2.1 | 0.6 |  | 44 | 3.0 | 0.3 |  | 68 | 4.3 | 0.9 |
| 21 | 2.2 | 0.4 |  | 45 | 3.0 | 0.2 |  | 69 | 4.4 | 0.5 |
| 22 | 2.2 | 0.3 |  | 46 | 3.0 | 0.2 |  | 70 | 4.7 | 0.4 |
| 23 | 2.2 | 0.3 |  | 47 | 3.1 | 0.5 |  | 71 | 4.9 | 0.5 |
| 24 | 2.3 | 1.1 |  | 48 | 3.1 | 0.8 |  | 72 | 7.9 | 0.3 |
|  |  |  |  |  |  |  |  | Mean | 2.6 | 0.5 |
